# Supplementary material for: Segmentally duplicated regulatory elements undergo human-specific rewiring
Source: bioRxiv. 2025 Dec 5:2025.12.03.692127. Preprint. [Version 1] doi: 10.64898/2025.12.03.692127 (PMC12822585; doi:10.64898/2025.12.03.692127)
Supplement: Supplementary file 1 [file NIHPP2025.12.03.692127V1-supplement-1.pdf]

bioRxiv preprint doi: https://doi.org/10.64898/2025.12.03.692127; this version posted December 5, 2025. The copyright holder for this preprint (which was not certified by peer review) is the author/funder, who has granted bioRxiv a license to display the preprint in perpetuity. It is made available under a CC-BY 4.0 International license.

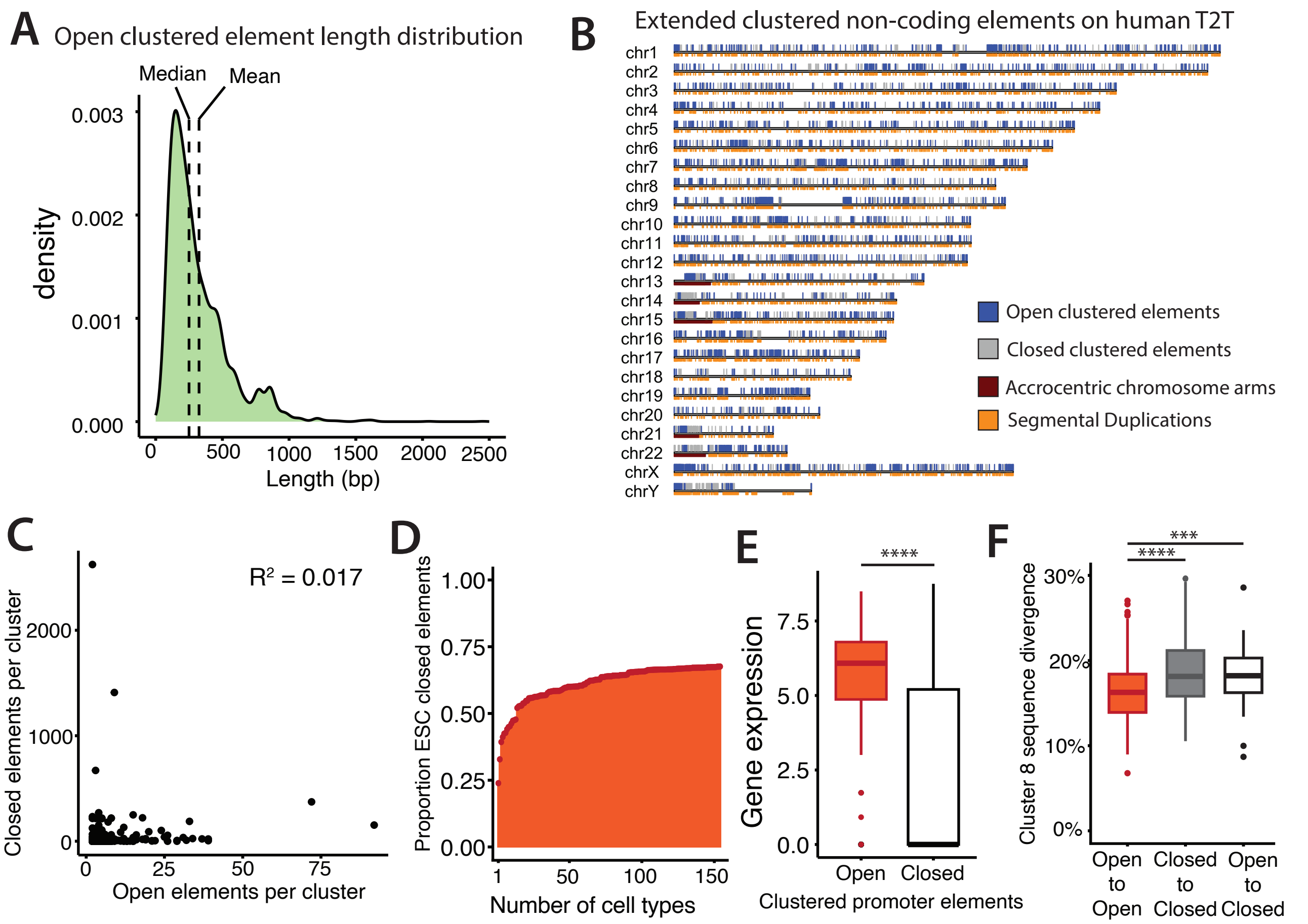

Figure S1. Open and closed clustered elements harbor differences in ESCs. (A) Location of embryonic stem cell (ESC) open clustered elements, closed clustered elements, segmental duplications and acrocentric chromosome arms in the human T2T reference genome. **(B)** Size distributions of open ESC clustered elements Eleven elements larger than 2500 bp were filtered out for ease of visualization. **(C)** Correlation of number of open and closed ESC elements per cluster. **(D)** Proportion of ESC closed elements that have accessible chromatin in other ENCODE cell types as increasing cell types are considered. **(E)** Comparison of gene expression between gene with open vs closed ESC promoters. Y-axis values reflect Log10-transformed fragments per kilobase plus a pseudocount. (Wilcoxon; ****: $p < 0.0001$). **(F)** Percent divergence between extended Cluster 8 elements, comparing sequence divergence between open elements, between open and closed elements, and between closed elements. (t-test; ***: $p < 0.001$, ****: $p < 0.0001$)

bioRxiv preprint doi: https://doi.org/10.64898/2025.12.03.692127; this version posted December 5, 2025. The copyright holder for this preprint (which was not certified by peer review) is the author/funder, who has granted bioRxiv a license to display the preprint in perpetuity. It is made available under a CC-BY 4.0 International license.

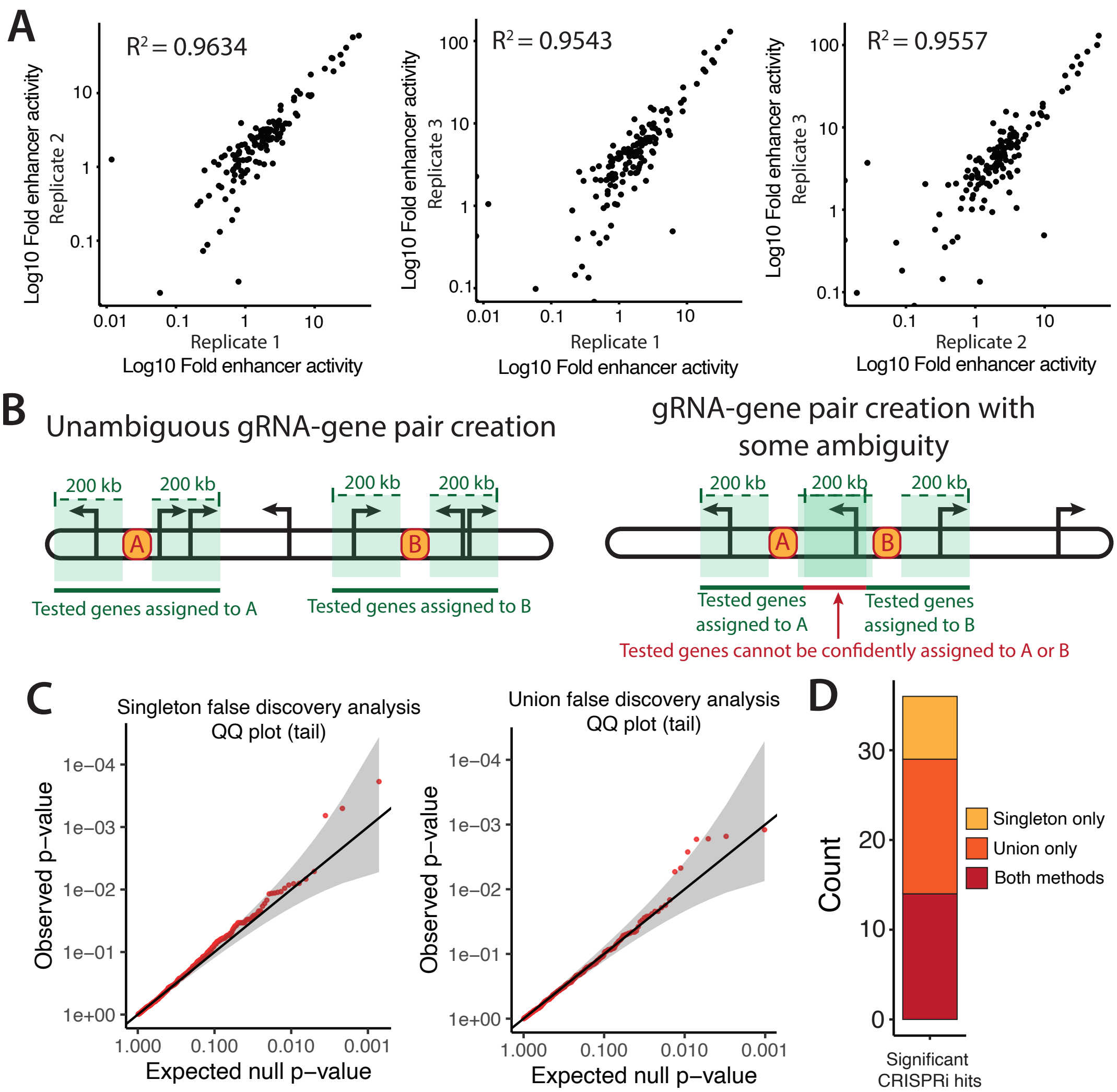

Figure S2. Functional data analysis quality (A) Correlation of three STARR-seq replicates performed in H9 ESCs. Axes are Log10 transformed **(B)** Representation of gRNA-gene pair creation for association testing by sceptre with a 200kb distance filter, where elements "A" and "B" are paralogous in a cluster. Arrows denote location of genes in the model. **(C)** Quality-control false discovery QQ plots produced by sceptre for both singleton and union analyses. No false positives were detected in either analysis. **(D)** Number of significant clustered element CRISPRi hits that were found in the singleton analysis, the union analysis or both analysis methods.

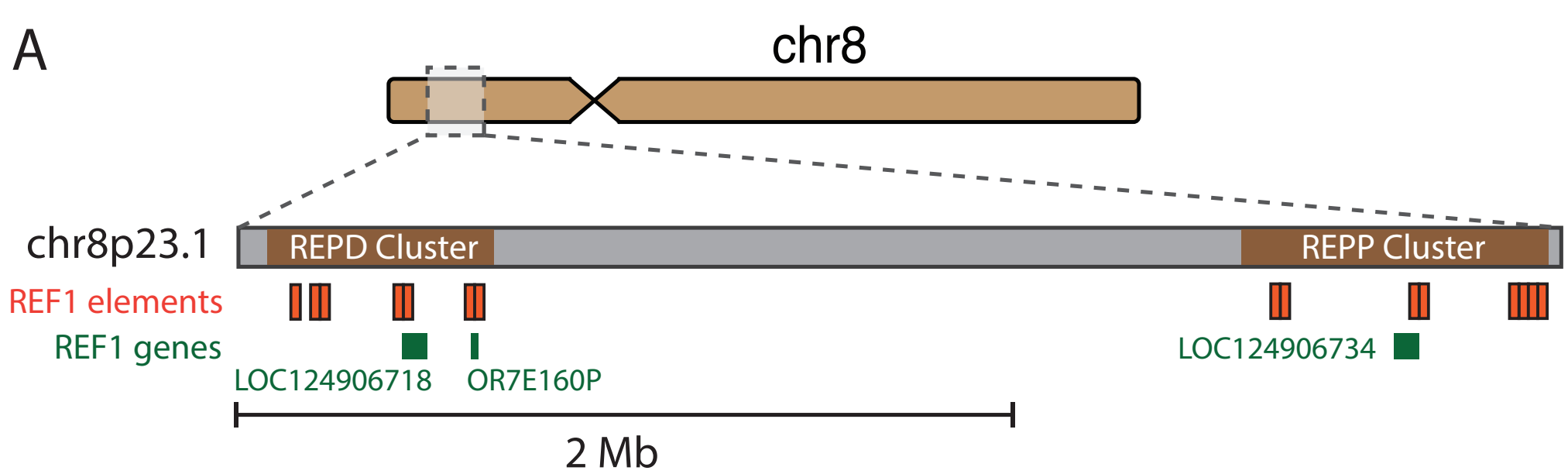

Figure S3. Chromosome 8 SD clusters harbor REF1 promoters (A) Graphical representation of REF1 elements within segmental duplication clusters on chromosome 8p23.1.

bioRxiv preprint doi: https://doi.org/10.64898/2025.12.03.692127; this version posted December 5, 2025. The copyright holder for this preprint (which was not certified by peer review) is the author/funder, who has granted bioRxiv a license to display the preprint in perpetuity. It is made available under a CC-BY 4.0 International license.

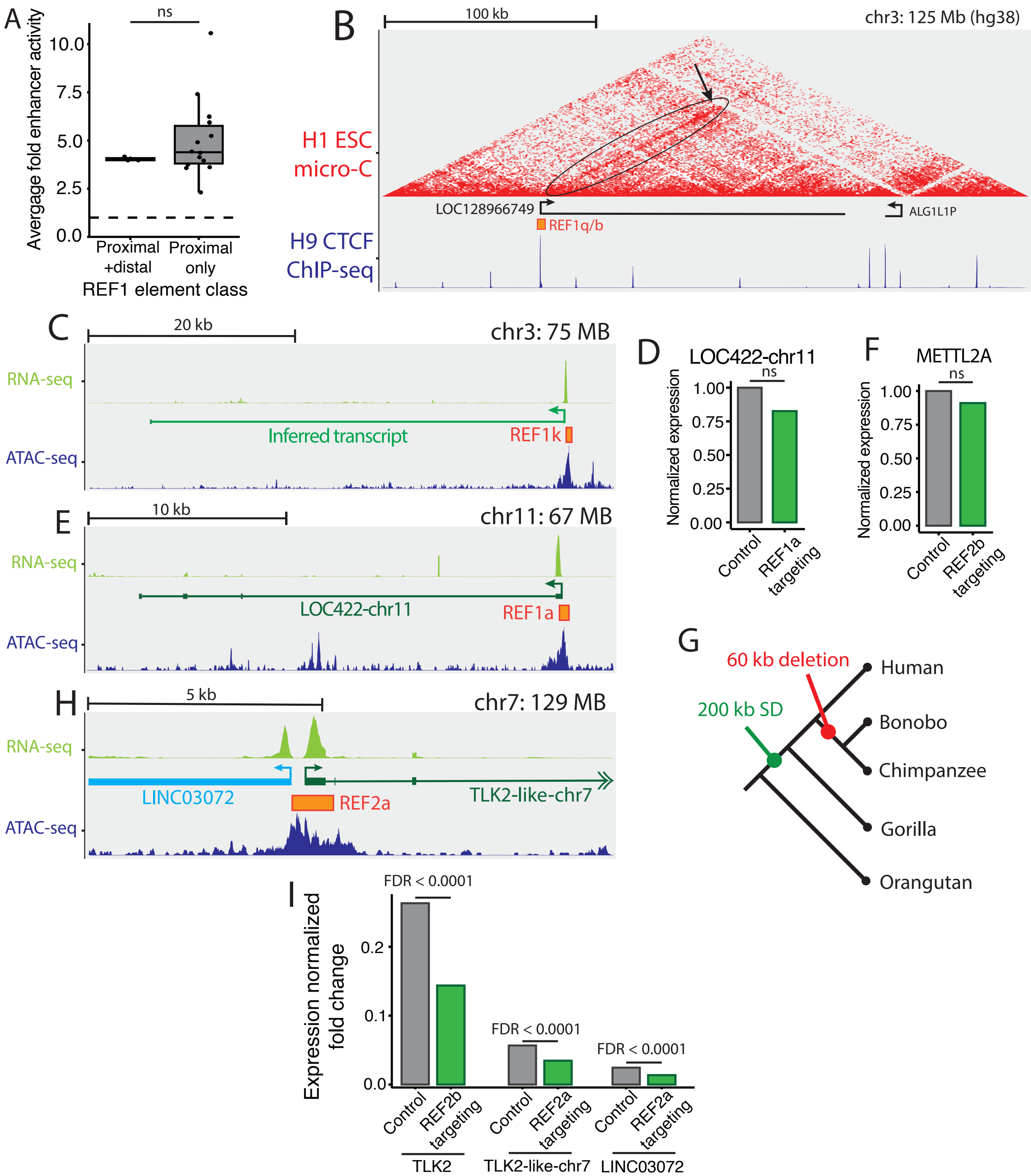

Figure S4. Element class flexibility associated data (A) Comparison of enhancer activity between CRISPRi-targeted REF1 elements that had proximal and distal regulatory activity compared to REF1 elements that had proximal activity only, measured by STARR-seq (Wilcoxon test, ns: $p > 0.05$). **(B)** 3D genome organization of the *LOC128966749-ALG1L1P* promoter-promoter interaction visualized on the hg38 genome assembly. The oval shows the stripe domain facilitated by the *LOC128966749* promoter, and the arrow shows the interaction with the *ALG1L1P* promoter. **(C)** Genomic context for REF1k. A transcription start site was inferred based on 5'-capture RNA-seq from ESCs and a gene model lifted from the hg38 assembly. **(D)** Normalized *LOC422-chr11* expression upon repression of REF1a with CRISPRi (ns: Benjamini-Hochberg FDR > 0.1) **(E)** Genomic context for REF1a. *LOC422-chr11* was lifted from the hg38 reference. **(F)** Normalized *METTL2A* expression upon repression of REF2b with CRISPRi (ns: Benjamini-Hochberg FDR > 0.1). **(G)** Primate phylogeny with arbitrary branch lengths showing the duplication history of the segmental duplication that created REF2a. **(H)** Genomic context of REF2a. Genes *TLK-chr7* and *LINC03072* lifted onto the human T2T assembly from hg38. **(I)** Fold-change gene expression plots showing conserved regulatory interactions when REF2 elements are repressed with CRISPRi. Control and targeting gene expression bars are scaled by gene expression in all cells that did not receive a targeting gRNA, as calculated by Seurat (Benjamini-Hochberg FDR < 0.1).

bioRxiv preprint doi: https://doi.org/10.64898/2025.12.03.692127; this version posted December 5, 2025. The copyright holder for this preprint (which was not certified by peer review) is the author/funder, who has granted bioRxiv a license to display the preprint in perpetuity. It is made available under a CC-BY 4.0 International license.

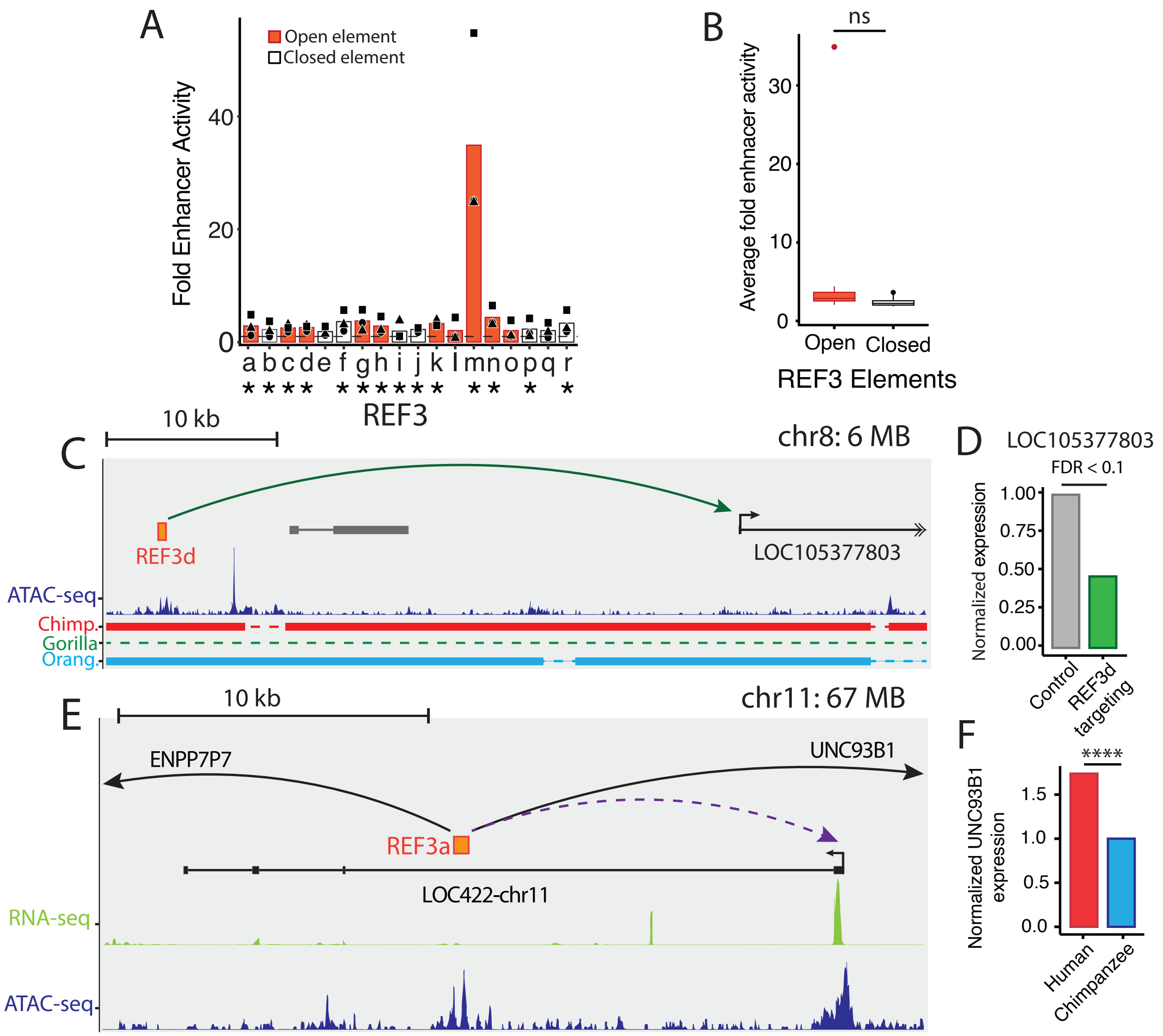

Figure S5. REF3 rewiring associated data (A) Fold enhancer activity of REF3 elements over negative controls. Data point shape denotes replicate number. Dotted line is the average of negative controls (Stauffer's method, *: $p < 0.01$). **(B)** Comparison of fold enhancer activity in REF3 between open and closed elements (Wilcoxon; ns: $p > 0.5$). **(C)** Genomic context for REF3d. Curved arrows represent a distal regulatory connection. **(D)** Normalized *LOC105377803* expression upon CRISPRi repression of REF3a (Benjamini-Hochberg FDR > 0.1) **(E)** Genomic context of REF3a. The *LOC422-chr11* gene model was lifted onto human T2T from hg38. Curved arrows represent distal regulatory interactions. **(F)** Human *UNC93B1* expression normalized to chimpanzee *UNC93B1* expression. Data from *Gokhman et. al., 2021*, using human-chimpanzee allotetraploid cells (Differential expression test; $p < 0.0001$: ****).

bioRxiv preprint doi: https://doi.org/10.64898/2025.12.03.692127; this version posted December 5, 2025. The copyright holder for this preprint (which was not certified by peer review) is the author/funder, who has granted bioRxiv a license to display the preprint in perpetuity. It is made available under a CC-BY 4.0 International license.

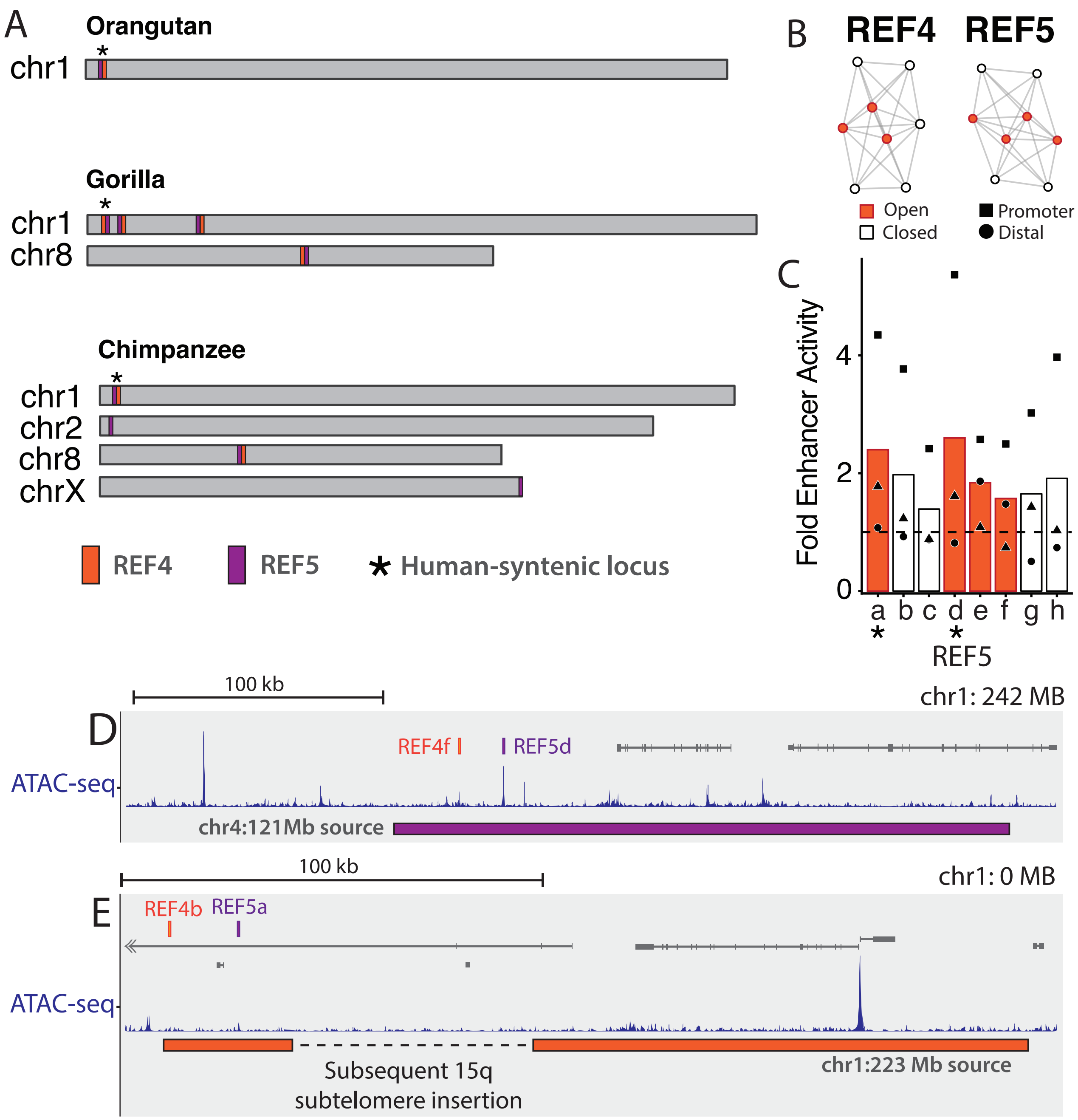

Figure S6. Lineage-specific duplications of REF4 and REF5. **(A)** Locations of REF4 and REF5 elements in T2T primate genomes. **(B)** REF4 and REF5 visualized with edge lengths corresponding to percent divergence between noncoding element nodes. **(C)** Fold enhancer activity of REF5 elements over negative controls, measured by STARR-seq. Data point shape denotes replicate number. Dotted line is the average of negative controls (Stauffer's method, *: $p < 0.01$). **(D-E)** Genomic context showing the source sequence for human-specific SDs containing REF4/5 elements.
